# Bioinformatics Analysis of Actin Molecules: Why Quantity Does Not Translate Into Quality?

**DOI:** 10.3389/fgene.2020.617763

**Published:** 2020-12-10

**Authors:** Anna V. Glyakina, Oxana V. Galzitskaya

**Affiliations:** ^1^Institute of Protein Research, Russian Academy of Sciences, Pushchino, Russia; ^2^Institute of Mathematical Problems of Biology RAS, Keldysh Institute of Applied Mathematics, Russian Academy of Sciences, Pushchino, Russia; ^3^Institute of Theoretical and Experimental Biophysics, Russian Academy of Sciences, Pushchino, Russia

**Keywords:** monomer, filamentous, polymorphism, actin, resolution, charge

## Abstract

It is time to review all the available data and find the distinctive characteristics of actin that make it such an important cell molecule. The presented double-stranded organization of filamentous actin cannot explain the strong polymorphism of actin fibrils. In this work, we performed bioinformatics analysis of a set of 296 amino acid actin sequences from representatives of different classes of the Chordate type. Based on the results of the analysis, the degree of conservatism of the primary structure of this protein in representatives of the Chordate type was determined. In addition, 155 structures of rabbit actin obtained using X-ray diffraction analysis and electron microscopy have been analyzed over the past 30 years. From pairwise alignments and the calculation of root-mean-square deviations (RMSDs) for these structures, it follows that they are very similar to each other without correlation with the structure resolution and the reconstruction method: the RMSDs for 11,781 pairs did not exceed 3 Å. It turned out that in rabbit actin most of the charged amino acid residues are located inside the protein, which is not typical for the protein structure. We found that two of six exon regions correspond to structural subdomains. To test the double-stranded organization of the actin structure, it is necessary to use new approaches and new techniques, taking into account our new data obtained from the structural analysis of actin.

## Introduction

Actin was discovered in 1948 by the Hungarian biochemist Bruno Straub. This protein was named for its ability to activate (hence actin) ATP hydrolysis catalyzed by myosin. Actin is a muscle tissue protein, the polymerized form of which (F-actin) forms microfilaments—one of the main components of the cytoskeleton of eukaryotic cells. Actin makes up 5–15% of the total cellular protein and is the most important protein in eukaryotic cells (Lodish et al., 2000). Actin analogs have also been found in bacteria (Popp et al., 2008, 2010; Galkin et al., 2009) and archaea (Izoré et al., 2014; Braun et al., 2015). Actin monomer (G-actin) is a water-soluble globular structural protein with a molecular weight of 42 kDa, consisting of 375 or 374 amino acid residues. Differences in the amino acid sequences, both within the same species and between species, are extremely insignificant, no more than 25 amino acid substitutions. In vertebrates, depending on the isoelectric point, three actin isoforms are distinguished, α, β, and γ (Vandekerckhove and Weber, 1978). α-actin is mainly characteristic of muscle cells, while β- and γ-actin are characteristic of non-muscle cells. α-actin, in turn, is divided into three types: smooth muscle α-actin, α-actin of skeletal muscle and α-actin of cardiac muscle (Vandekerckhove and Weber, 1978; Gunning et al., 1983; Erba et al., 1988; Miwa et al., 1991).

**FIGURE 1:**
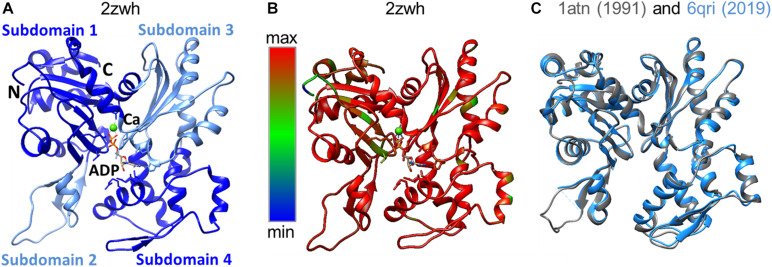
**(A)** 3D structure of α-actin. Actin-bound ADP molecule and Ca2 + cation are shown. **(B)** Conservation of the actin amino acid sequences of representatives of different classes of chordates was built by the Chimera program (Pettersen et al., 2004). Highly conserved regions of the polypeptide chain are colored by red, non-conserved regions by blue. **(C)** Spatial alignment of two rabbit actin structures deposited in the Protein Data Bank in 1991 and 2019.

Domains have historically been divided into large and small, although their sizes are almost the same. The N- and C-termini of the polypeptide chain are located in a small domain. Each of the domains has two subdomains. By definition, subdomain 1 (residues 1-32, 70-144, and 338-372) and subdomain 2 (residues 33-69) are part of a small domain. The large domain consists of subdomain 3 (residues 145–180 and 270–337) and subdomain 4 (residues 181–269) (Selby and Bear, 1956). The domains are separated by a deep cleft (Figure 1A). The actin monomer is rather flat and fits into a “parallelepiped” with dimensions of 55 Å × 55 Å × 35 Å.

Actin is one of the main components of myofibrils and, together with myosin and titin, provides muscle contraction. In other types of cells, actin forms a system of microfilaments and, together with other filamentous structures (microtubules and intermediate filaments), makes up the cytoskeleton and performs various functions (movement, cell reshaping, cytokinesis, exo- and endocytosis, redistribution of surface receptors, and other processes) (Carlier et al., 1997; Silacci et al., 2004; Kovar et al., 2006; Ferron et al., 2007).

The actin polymerization and depolymerization process is regulated by special proteins. For example, profilin, forming a complex with globular actin, prevents actin polymerization. Cytochalasin D binds to actin and forms a kind of “cap” at one end of the polymerizing actin, thereby regulating the polymerization process. There are proteins (latrunculin A) that prevent the polymerization of globular actin and proteins that “cut” actin filaments into short fragments. Conversely, there are proteins that “cross-link” already formed actin filaments, thus forming ordered rigid bundles of actin filaments or flexible coarse networks (Carlier et al., 1997; Silacci et al., 2004; Kovar et al., 2006; Ferron et al., 2007).

Actin monomers can interact with each other to form F-actin. The polymerization process can be initiated by increasing the concentration of cations or by adding special proteins. The polymerization process becomes possible because actin monomers can recognize each other and form intermolecular contacts. *In vitro*, at physiological salt concentrations, G-actin polymerizes into filamentous F-actin (Page et al., 1998; Oda et al., 2009; Dominguez and Holmes, 2011; Thomasson and Macnaughtan, 2013).

Historically examining all the structural work of actin organization to find direct evidence of double-stranded actin organization, we have concluded that there is no direct evidence for the existence of a double helix. The authors of the work on X-ray diffraction could not give an unambiguous answer about the double helix organization of actin (Selby and Bear, 1956), and only the authors of the work (Hanson and Lowy, 1963), based on electron microscopy data, taking into account the data of the previous X-ray work, said that actin most likely has a double helix organization. Apparently, such a conclusion was nevertheless dictated by the recent discovery of the DNA double helix at that time. Over time, “the highly-likely model” became generally accepted and entered all textbooks (Jegou and Romet-Lemonne, 2020). A lot of data have been accumulated that is not suitable for the proposed organization. This model, on the one hand, cannot explain the strong polymorphism of filamentous actin, but, on the other hand, does not contradict the picture of interaction with partners, since the interaction sites are located on one side of the actin molecule. The aim of this work is to obtain more structural information about such important molecule as actin, to validate the structure of F-actin.

## Materials and Methods

### Databases

A set of 296 amino acid sequences of actin from representatives of different classes of the Chordate type: mammals (*Homo sapiens*, *Bos taurus*, *Mus musculus*, *Rattus norvegicus*); aves (*Gallus gallus*, *Anas platyrhynchos*, *Meleagris gallopavo*); Reptiles (*Chelonia agassizi*, *Pelodiscus sinesis*, *Anolis carolinensis*); amphibian (*Xenopus tropicalis*); fish (*Danio rerio*, *Tetraodon nigrovoridis*, *Oryzias latipes*) were taken from the UniProt database.

A list of 155 protein structures of actin was taken from the UniProtKB database, record number P68135, gene ACTA1, wild rabbit species (*Oryctolagus cuniculus*). These structures were deposited in the Protein Data Bank between 1991 and early 2020. Half of these structures (72) are actin monomeric structures, the other half (83) are structures containing two or more actin monomers.

The canonical reviewed protein sequences (20,364 fasta records) were extracted from the human reference proteome (uniprot request reviewed:yes AND organism: “Homo sapiens (Human) [9606]” AND proteome:up000005640).

### Structural Characteristics

Spatial alignment of 155 actin structures, calculation of the root-mean-square deviation (RMSD) of Cα atoms for each pair of superimposed structures, and calculation of the accessible surface area (ASA) for each amino acid residue in actin structures were performed using the YASARA program (Krieger et al., 2002). If pdb file had several actin structures, then only one (first) structure was taken for spatial alignment and RMSD calculation. ASA was defined as the surface area over which a water ball with a radius of 1.4 Å rolls. A residue was called external if its ASA was more than 50% of the maximum ASA observed in the Protein Data Bank for each type of amino acid residue.

## Results and Discussion

### Conservation and Splicing Sites

The amino acid sequence of skeletal and cardiac muscle actin consists of 375 amino acid residues, including one unusual amino acid residue, 3-methylhistidine, which is formed post-translationally. The N-terminal amino acid of actin is acetylated. According to the results of the alignment of the actin amino acid sequences, a high degree of conservatism of the primary structure of actin was observed (Figure 1B). The spatial alignment of the two rabbit actin structures resolved in 1991 and 2019 is presented in Figure 1C. The RMSD for this pair is 0.6 Å, where the percent of amino acid residues aligned is 94%.

Cytoplasmic actins differ from vertebrate skeletal muscle actin only by 25 substitutions. It is essential that the region of the polypeptide chain containing residues 18–75 is stable, while regions 2–18 and 259–298 contain many substitutions. The high conservatism of the primary structure of actin, apparently, is a consequence of its high functional activity, which requires the preservation of the centers of interaction with both other actin molecules and actin-binding proteins. It should be noted that regions 18–25 and 259–298 do not belong to the F-actin core (Glyakina et al., 2020).

The data on the primary structure of actin in higher plant obtained on the basis of the analysis of nucleotide sequences of actin genes indicate that the variability of plant actin is much higher than that of animal actin. In particular, soybean isoactins contain 35–45 substitutions. In general, plant actin differs from animal actin by 55–65 amino acid residues. Actin substitutions in plants include significant number of charged residues; therefore, their isoelectric point can differ by almost one unit (pH 5.1–5.8) (Meagher and McLean, 1990).

Although more than 95% of the known protein sequences are derived from DNA translation, there is no single reference nucleic acid sequence for the given UniProtKB/Swiss-Prot protein sequence. To obtain splicing sites, we aligned the nucleotide sequence of human actin to the corresponding gene. There are only three substitutions in amino acid sequences between the human and rabbit actin sequences. Therefore, the splicing sites for the human gene will coincide with the splicing sites of the rabbit actin gene (Supplementary Figure S1).

**FIGURE 2:**
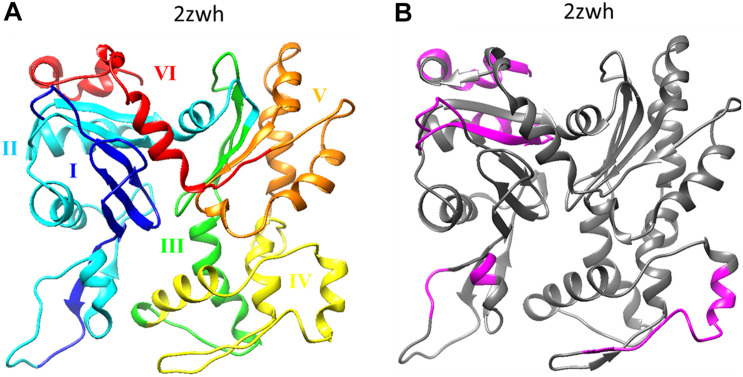
**(A)** Six exons of α-actin are colored on the 3D structure: I (residues 1–42) exon is colored by blue, II (residues 43–151) – cyan, III (residues 152–204) – green, IV (residues 205–269) – yellow, V (residues 270–329) – orange, I (residues 330–375) – red. **(B)** Disordered regions (1–8, 51–54, 58–60, 101–120, 228–243, 365–377) calculated using the program IsUnstract (http://bioinfo.protres.ru/IsUnstruct/) (Lobanov et al., 2013).

If we compare the 3D actin structure with the splicing sites, we can see that exon IV (residues 205–269) is included in subdomain 4 (residues 181–269), and exon V (residues 270–329) is part of subdomain 3 (residues 270–337) (see Figures 1A, 2A).

### Amino Acid Composition

To assess the increased and decreased content of one or another amino acid in the composition of α-actin from the skeletal muscles of rabbit, a comparison with the mean proteomic values of amino acid composition was carried out.

There is an increased content of amino acids such as Ile, Met, Thr, Tyr, and a decreased content of Cys, Leu, Gln compared to the average proteomic values for human, which are taken as a unit (Supplementary Figure S2).

Rabbit actin contains five cysteine residues (Cys10, Cys217, Cys257, Cys285, and Cys374). Smooth muscle actin contains another cysteine residue at position 17. Non-muscle actin contains two additional cysteine residues, Cys17 and Cys272. However, Cys10 is replaced by Val10 in actin, and the total number of cysteine residues is six. Only one of these residues, Cys374, is exposed on the surface of an intact ATP containing G-actin molecule. The availability of other cysteine residues is determined by the degree of nativeness of the molecule and the type of nucleotide. In a solution with a high concentration of ADP, in addition to Cys374, another cysteine residue, apparently, Cys10, becomes available for SH-reagents. However, there are no disulfide bonds in the structure of rabbit actin.

### Characteristics of Actin Surface: Distribution of Charges and Ligand Sites

The distribution of charged amino acid residues in rabbit actin is shown in Supplementary Figure S3. The charged amino acid residues make up 24%: the number of Arg is 18, Lys – 19, Glu – 28, Asp – 22. Therefore, the charge of the G-actin molecule is negative (−13). The N-terminal segment of α-actin contains four acidic amino acid residues Asp-Glu-Asp-Glu, in the N-terminal segment of β- and γ-isoactins there are only three Asp and three Glu in β- and γ-isoactin, respectively. Substitutions of amino acid residues in the N-terminal segment of the polypeptide chain significantly affect the total charge of the molecule, changing the actin isoelectric point in the pH range 5.4–5.5. When analyzing the amino acid sequence of actin, attention is drawn to a large number of negatively charged groups, especially at the N-terminus of the chain. So, out of five N-terminal amino acid residues, four contain carboxyl groups in the side chains, and among the first 25 amino acid residues, seven are negatively charged.

Oztug Durer et al. (2010) try to investigate the constrains in D-loop (residues 39–50) plasticity as determined by its interactions with other dynamic elements of actin, including the C-terminus, the W-loop (residues 165–172), and the H-loop (residues 264–273). The involvement of these structural loops in contacts between monomers in the actin filament was predicted by the models of double-stranded organization of F-actin. The authors showed that introduction of disulfide bonds between residues 45, 47, 50 (D-loop), and residue 169 (W-loop) or 265 (H-loop) leads to the disruption of F-actin structure, which is expressed in the appearance of amorphous aggregates in the electron microscopy images (Oztug Durer et al., 2010). Consequently, the double-stranded model of F-actin maybe not the only one.

The fraction of charged amino acid residues of each type (Arg, Lys, Glu, Asp) in rabbit actin and bovine p450 (comparable to the size of actin, 475 amino acid residues) is shown in Supplementary Table S1. The fractions of charged amino acid residues in these proteins are practically the same and are close to the proteomic values (Supplementary Table S1 and Supplementary Figure S2). However, most of the charged amino acid residues in rabbit actin are found within the protein as compared to bovine p450.

Actin is a unique building material widely used by the cell to construct various elements of the cytoskeleton and contractile apparatus. This is due to the fact that the processes of actin polymerization and depolymerization can be easily regulated using special proteins that bind to actin. Thus, actin is involved in many protein–protein interactions. The number of interaction partners for yeast actin is 222 according to the STRING database (Szklarczyk et al., 2015) (Supplementary Figure S4). There are no interaction partners for human actin in this database.

**FIGURE 3:**
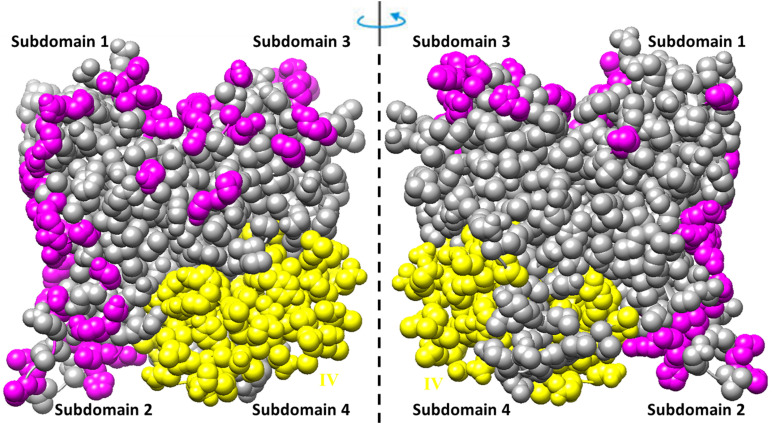
Amino acid residues in the rabbit actin structure (2zwh) interacting with more than one of the 11 ligands (actin, profilin, gelsolin, DBP, cofilin, DNaseI, myosin, leiomodin, fimbrin, vinculin, tropomyosin) (Tikhomirova et al., 2018) are colored by magenta. Exon IV is colored by yellow.

The residues involved in the interactions with more than one of the ligands (actin, profilin, gelsolin, DBP, cofilin, DNaseI, myosin, leiomodin, fimbrin, vinculin, tropomyosin) (Tikhomirova et al., 2018) are shown in Figure 3. Interestingly, that such residues are located predominantly on one side of the molecule and are not located in subdomain 4, which includes exon IV (residues 205–269) (see Figures 2A, 3 and Supplementary Figure S1).

### Structural Alignments

Of the 155 three-dimensional (3D) structures of rabbit actin, 72 are monomeric and 83 are oligomeric. 100 structures were obtained by X-ray diffraction analysis with a resolution of 1.29–7.88 Å, 52 structures by cryo-electron microscopy with a resolution of 3.6–70 Å, and one structure by fiber diffraction with a resolution of 3.3 Å, and two model structures (1ALM and 1UY5).

We performed pairwise spatial alignments of 154 actin structures and calculated the RMSDs between the Cα atoms. The RMSDs for 11,781 pairs do not exceed 3 Å: 0–1 Å for 5461 pairs, 1–2 Å for 5522 pairs, and 2–3 Å for 798 pairs (Supplementary Figures S5A,C). The fraction of aligned amino acid residues in each pair is more than 50% (see Supplementary Figure S5B). It should be noted that the 3D structures of the actin monomer are very similar. It turns out that for almost 30 years the quality of the obtained actin structures has not improved, despite new technologies.

**FIGURE 4:**
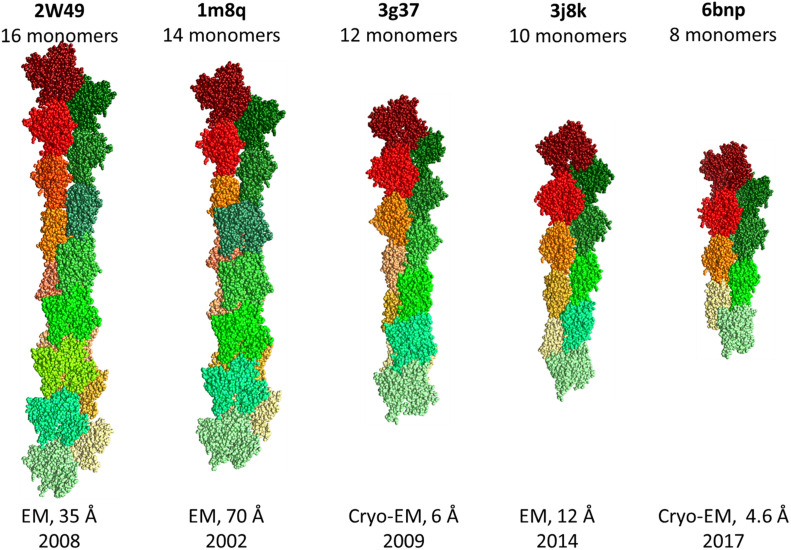
Structures of F-actin from the Protein Data Bank.

It should be noted that the RMSD between monomers in the structures of filamentous actin does not exceed 1.3 Å (Figure 4). It is very strange that the RMSD is zero for monomeric structures 2W49 and 1M8Q, since the actin structure contains flexible/disordered regions that will add polymorphism to the structural organization of actin (Figure 2B) (Lobanov et al., 2013).

## Conclusion

Bioinformatics analysis of actin showed that:

(1) The amino acid sequences of actin in representatives of different classes of chordates are highly conservative;
(2) Analysis of exons showed that exon IV (residues 205–269) corresponds to subdomain 4 (residues 181–269) and exon V (residues 270–329) corresponds to subdomain 3 (residues 270–337);
(3) The 3D actin rabbit monomer structures resolved from 1991 to 2020 (during 30 years) are very similar: the RMSD for 11,781 pairs does not exceed 3 Å, the RMSD is about zero for monomeric structures in the filamentous actin (2W49 and 1M8Q);
(4) Most of the charged amino acid residues are located within actin structure, which is unusual for a protein structure.

Due to the high polymorphism, it has not yet been possible to obtain the structure of filamentous actin using X-ray diffraction analysis. Thus, all hope for obtaining this structure is for new methods such as XFEL and cryo-electron microscopy. For further reconstruction of filamentous actin using cryo-electron microscopy, it is necessary to take into account the adjustment of monomers in the organization of filament, rather than simple copying of the “building block,” which gives a close to zero RMSD between monomers, which is observed for some filament structures from the Protein Data Bank.

## Data Availability Statement

The original contributions presented in the study are included in the article/Supplementary Material. Further inquiries can be directed to the corresponding author.

## Author Contributions

OG conceived and supervised the study. AG implemented the calculations, drew figures, and funded the manuscript. AG and OG analyzed the data and wrote the manuscript. Both authors read and approved the final version of the manuscript.

## Conflict of Interest

The authors declare that the research was conducted in the absence of any commercial or financial relationships that could be construed as a potential conflict of interest.
