# Supplementary material for: Bioinformatics Analysis of Actin Molecules: Why Quantity Does Not Translate Into Quality?
Source: Front Genet. 2020 Dec 10;11:617763. doi: 10.3389/fgene.2020.617763 (PMC7758494; doi:10.3389/fgene.2020.617763)
Supplement: Supplementary file 1 [file Data_Sheet_1.docx]

Bioinformatics Analysis of Actin Molecules: Why Quantity Does not Translate into Quality?

Anna V. Glyakina^1,2^, Oxana V. Galzitskaya^1,3*^

^1^ Institute of Protein Research, Russian Academy of Sciences, 142290, Pushchino, Russia

^2^ Institute of Mathematical Problems of Biology RAS, Keldysh Institute of Applied Mathematics of Russian Academy of Sciences, Pushchino, Russia

^3^ Institute of Theoretical and Experimental Biophysics, Russian Academy of Sciences, 142290 Pushchino, Russia

*** Correspondence:**Oxana V. Galzitskaya
ogalzit@vega.protres.ru


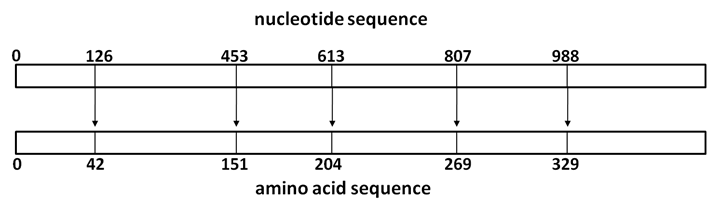


**Figure S1.** Structure of gene of human actin. 6 exons, 5 splicing sites (https://www.ncbi.nlm.nih.gov/nuccore/NM_001100.4; <https://rest.ensembl.org/sequence/id/ENSG00000143632?content-type=text/x-fasta;multiple_sequences=true;type=cds;format=fasta>).


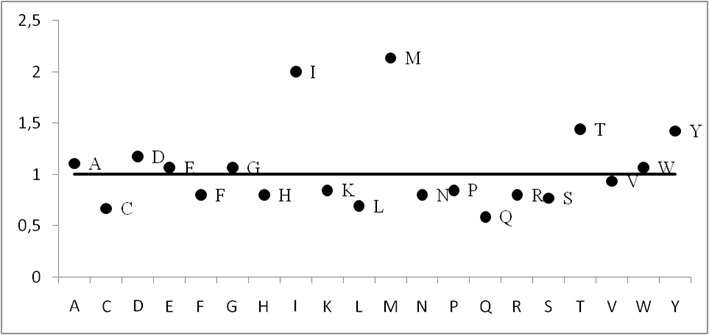


**Figure S2.** Comparison of the amino acid composition of rabbit actin with the mean human proteomic values (black line). The frequencies of amino acids for actin were normalized to the frequencies of amino acids for the human proteins.


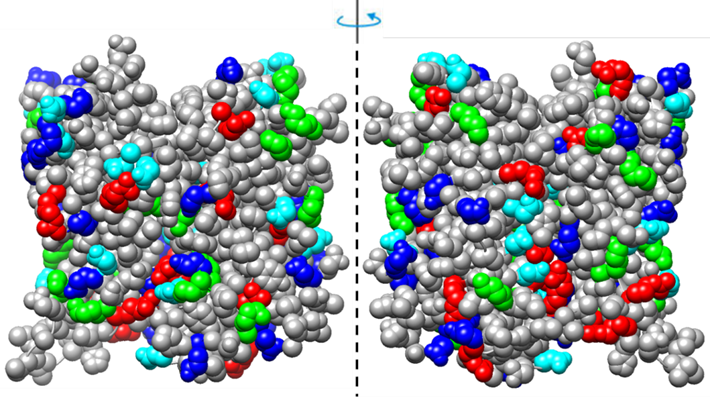


**Figure S3.** Charged amino acid residues in the actin structure (2zwh) are colored: Arg – red, Lys – green, Glu – blue, Asp – cyan.


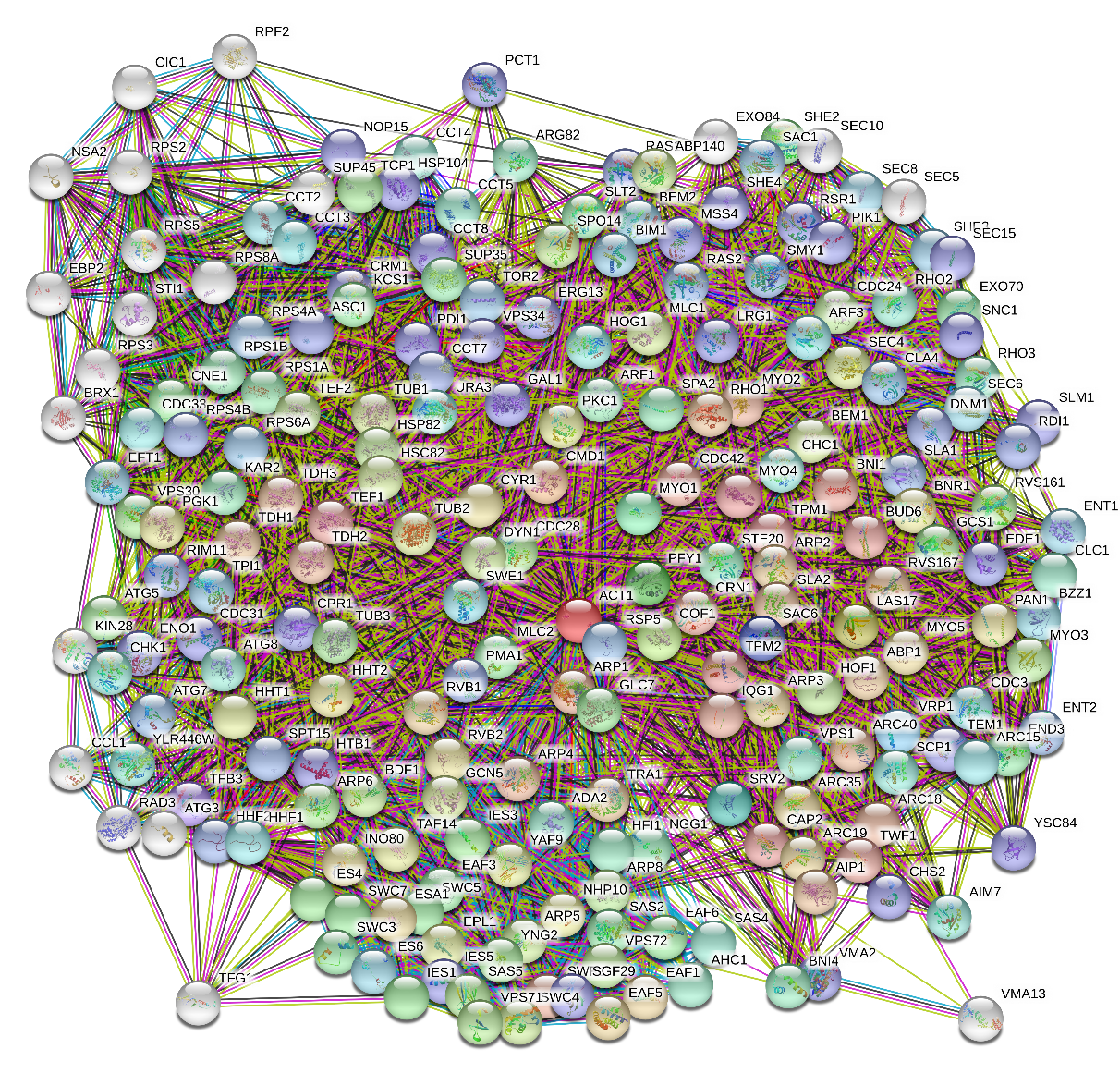


**Figure S4.** Network of interaction partners for yeast actin according to the STRING database. The number of partners is 222.


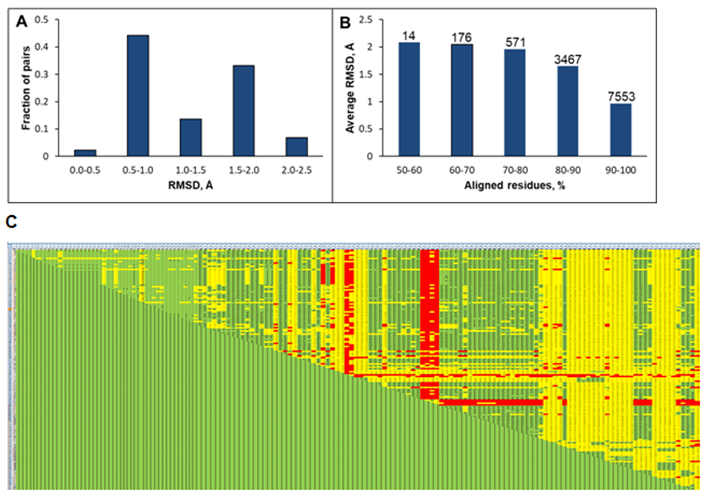


**Figure S5. (A)** RMSD distribution of the aligned pairs of rabbit actin structures. **(B)** The average RMSD for different fraction of aligned amino acid residues in each pair. **(C)** RMSD for 11781 pairs of aligned actin structures (green for RMSD < 1Å, yellow for 1Å ≤ RMSD < 2Å, red for 2Å ≤ RMSD < 3Å).

**Table S1. Fraction of all and external charged amino acid residues in rabbit actin (2zwh) and bovine p450 (3mzs)**

|  | Actin (2zwh) | | p450 (3mzs) | |
| --- | --- | --- | --- | --- |
|  | All, % | External, % | All, % | External, % |
| Lys | 5 | 11 | 6 | 21 |
| Arg | 5 | 11 | 7 | 42 |
| Glu | 7 | 18 | 7 | 23 |
| Asp | 6 | 14 | 5 | 9 |
| Cys | 1 | 0 | 0.4 | 0 |
